# Varietal Dependence of GLVs Accumulation and LOX-HPL Pathway Gene Expression in Four *Vitis vinifera* Wine Grapes

**DOI:** 10.3390/ijms17111924

**Published:** 2016-11-23

**Authors:** Xu Qian, Xiao-Qing Xu, Ke-Ji Yu, Bao-Qing Zhu, Yi-Bin Lan, Chang-Qing Duan, Qiu-Hong Pan

**Affiliations:** 1 Center for Viticulture & Enology, College of Food Science and Nutritional Engineering, China Agricultural University, Beijing 100083, China; qxhanyi@163.com (X.Q.); xuxiaoqing@outlook.com (X.-Q.X.); yukeji@cau.edu.cn (K.-J.Y.); clanyibin@gmail.com (Y.-B.L.); chqduan@cau.edu.cn (C.-Q.D.); 2 Department of Food Science and Engineering, College of Biological Sciences and Technology, Beijing Forestry University, Beijing 100083, China; Zhubaoqing@gmail.com

**Keywords:** green leaf volatiles, grape variety, lipoxygenase-hydroperoxide lyase pathway

## Abstract

Variety is one of the major factors influencing grape and wine aromatic characteristics. Green leaf volatiles (GLVs), derived from lipoxygenase-hydroperoxides lyase (LOX-HPL) pathway, are important components for the aromatic quality of grapes and wines. However, the varietal difference regarding GLVs accumulation and related gene expression are poorly studied. This work exhibited that the accumulation of various GLVs and the expression of LOX-HPL pathway genes in four *Vitis vinifera* wine grape cultivars: Syrah, Muscat Tchervine, Gewürztraminer and Chardonnay. The results showed a variety dependence of GLVs profile. Muscat Tchervine harvested grapes contained less C6 aldehydes and the most abundant esters, which corresponded to very low *VvLOXA* and *VvHPL1* expression abundance as well as high *VvAAT* transcript in this variety. High expression level of both *VvLOXA* and *VvHPL1* paralleled with higher level of C6 aldehydes together with higher alcohols in Syrah grape. Gewürztraminer and Chardonnay grapes had high aldehydes and alcohols as well as low esters, which were resulted from their higher expression level of *VvLOXA* or *VvHPL1* and lower *VvAAT*. From these above corresponding relations, it is concluded that *VvLOXA*, *VvHPL1* and *VvAAT* in the LOX-HPL pathway are targets for altering GLVs composition in the grape varieties.

## 1. Introduction

Volatile organic compounds are vital for the quality of grape berries and wines. They determine the aromatic quality and variety characteristics. Hundreds of volatile organic compounds have been identified and they are divided into several major families: terpenoids, fatty acids derived compounds, aromatic aldehydes and alcohols, thiols and methoxypyrazines [1]. The aromatic characteristics of each grape variety are due to its unique combination of these compounds. Among these volatile compounds, C6 aldehydes, alcohols and esters are named as Green Leaf Volatiles (GLVs) based on their odor description [2]. The C6 aldehydes are the most abundant volatile components in grape berry [3,4]. In addition, C6 aldehydes and C6 alcohols are precursors of hexyl acetate in wines. Hexyl acetate in wines is found to be grape dependent and contribute to fruity character [5]. Hence, the level of C6 compounds in grape berries are closely related to odor sensory of the wines made from these fruits. The C6 volatile compounds of wines, except (*Z*)-3-hexen-1-ol and (*Z*)-3-hexenyl acetate, showed an increasing pattern with increasing sugar contents and delayed harvest dates of grape berries [6]. This pattern of C6 volatiles was in agreement with other grape volatile evolution during berry development [4,7]. 

The C6 aldehydes are generally derived from linoleic acid and linolenic acid by grape lipoxygenase (LOX), hydroperoxides lyase (HPL) and (*3Z*)-(*2E*) enal isomerase. The aldehydes are then reduced by alcohol dehydrogenase (ADH) to form C6 alcohols, which can be esterified into acetates. Two *13-LOXs* were cloned from Sauvignon Blanc and their transcript rapidly accumulated in response to mechanical wounding and the infection of *Botrytis cinerea* [8]. *VvHPL1* and *VvHPL2* were cloned from Cabernet Sauvignon berry and identified with different enzymatic functions [9]. Several VvADHs have been biochemically identified in grape berry [10]. There have been accumulating studies aiming at revealing the physiological functions of GLVs and the regulation of LOX-HPL pathway in plants [11,12,13,14]. The biological significance of this pathway has been well demonstrated to date. The GLVs are thought to be signals released from plants under biotic or environmental stress [15]. Therefore, the GLVs are not only important aroma compounds for both grape berry and wine, but also act as efficient defense chemicals.

Variety is considered to be one of the most important factors influencing grape and wine aromatic characteristics. The varietal aroma depends on the overall profile of odor-active compounds present in grapes and wines [16]. Wine grapes generally have been grouped into three categories based on the level of free monoterpenoids: Muscat varieties (high free monoterpene content), non-Muscat aromatic varieties (medium free monoterpene content) and neutral varieties (very low free monoterpene content) [7]. Terpenes and C13-norisoprenoids are demonstrated to be important contributors to the aroma of Muscat and non-Muscat aromatic varieties [17,18]. More than 50 terpenes have been identified as responsible for varietal flavor of Muscat grape varieties. In addition, C6 alcohols and C6 aldehydes are also considered as characteristic compounds of non-Muscat varieties such as Cabernet Sauvignon, Monastrell and Tempranillo [3]. Recently, C6 compound contribution to wine odor has been paid attention. For example, (*Z*)-3-hexen-1-ol and hexanol were considered as secondary sources of differences between young red wines from Grenache, Tempranillo, Merlot and Cabernet Sauvignon, and the byproducts of yeast amino acid metabolism were major different components [19]. A recent study compared the varietal differences between Shiraz and Cabernet Sauvignon grapes and wines with regard to volatile esters, and found that C6-acetates were the most importantly influenced compositions [20]. C6 compounds in grape berries were concluded to accentuate acetate synthesis in Shiraz wines due to higher nitrogen in grape juice, and a more active LOX-HPL pathway in Shiraz grape was suggested [20]. Thus it can be seen that the significance of C6 compounds of grape has been again established from the view of varietal typicality and the varietal differences in LOX-HPL pathway also cause great interest. However, the evolution of GLVs profiles and the expression of LOX-HPL pathway genes in developing berries from different types of grape varieties remain to be understood. 

Although the profiling of GLVs and their influencing factors in Cabernet Sauvignon and Merlot grapes and wines have been documented and discussed to some extent [4,21,22,23], the accumulation of these volatiles in other grape varieties remain unclear and the transcript level of responsible genes and their differences and similarities are to be assessed. In this paper, we examined the GLVs profiles of four different cultivars: Syrah (Syr, neutral red cultivar), Muscat Tchervine (MT, Muscat cultivar), Gewürztraminer (Gew, non-Muscat aromatic cultivar) and Chardonnay (Char, neutral white cultivar) in two consecutive vintages. All of them, which belong to various types of wine grapes and present dramatically different aromatic attributes, were cultivated in the same vineyard in Gansu province of the northwestern China with semi-arid climate. Furthermore, the relative expressional levels of LOX-HPL genes were revealed. Then we integrated the data of metabolic profiles and performance of the transcriptional level of LOX-HPL pathway to illustrate the varietal differences. The obtained results will add to the understanding of GLVs profiles of different types of grape varieties and aid winemakers to select the optimum grape varieties in the production of multi-varietal wines where “green leaf” odor could be targeted for enhancement or weakened to obtain a good balance of wine aroma.

## 2. Results and Discussion

### 2.1. Green Leaf Volatiles (GLVs) Profiling during Berry Development of Four Wine Grape Varieties 

**Figure 1:**
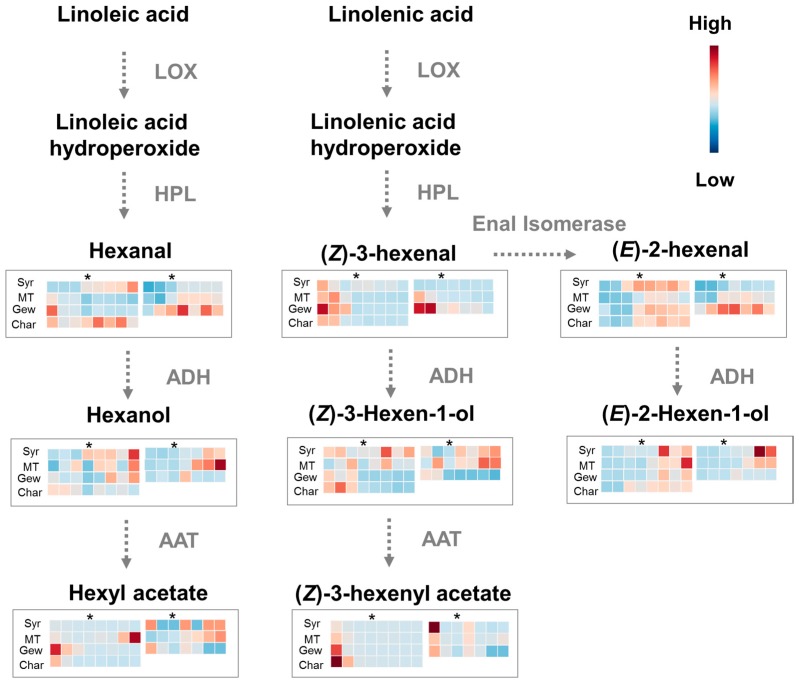
Heat maps of green leaf volatiles (GLVs) of different varieties in 2010 and 2011 vintages. The left of each colored square represent 17, 31, 43, 59, 73, 87, 101 and 106 days after flowering (DAFs) in 2010 vintage, and the right of each square represent 33, 48, 69, 75, 89, 102 and 112 DAFs in 2011 vintage, respectively. * indicates the veraison stage. LOX, lipoxygenase; HPL, hydroperoxides lyase; ADH, alcohol dehydrogenase; AAT, alcohol acyl transferases; Syrah (Syr, neutral red cultivar); Muscat Tchervine (MT, Muscat cultivar); Gewürztraminer (Gew, non-Muscat aromatic cultivar); Chardonnay (Char, neutral white cultivar).

Figure 1 displays a brief metabolic pathway graph, and the colored boxes represented the relative concentration of these compounds (red: higher level; blue: lower level). The data in each square of heat map were homogenized. Hexanal and (*E*)-2-hexenal are two major C6 aldehydes and they are the most abundant volatile compounds in grapes as known [4,7]. This investigation also revealed that the grapes had considerable concentrations of hexanal and (*E*)-2-hexenal, and a relatively low level of (*Z*)-3-hexenal (Table S1). (*E*)-2-Hexenal roughly increased during early berry development followed by a decrease at late ripening stage. A similar (*E*)-2-hexenal accumulating pattern was observed for these four varieties. This increase normally started at about 60 days after flowering (DAF) for Muscat Tchervine, Gewürztraminer and Chardonnay, but at about 45 DAF for Syrah berries in the 2010 vintage. On the contrary, Gewürztraminer berry started accumulating (*E*)-2-hexenal in a much earlier stage than Syrah and Muscat Tchervine in the 2011 vintage. Generally both Syrah and Gewürztraminer presented more rapid accumulation of (*E*)-2-hexenal than Muscat Tchervine. Regarding hexanal, its evolution pattern varied with varieties and vintages. The concentration of hexanal constantly increased during the developing stages of Syrah berries in both vintages. An upward trend was observed for hexanal during the berry development of 2011 vintage for both Muscat Tchervine and Gewürztraminer. However, the highest concentration of hexanal appeared at early stage in 2010 vintage for these two varieties, which was drastically different from other *Vitis vinifera* cultivars such as Cabernet Sauvignon [4] and Riesling [7]. It was speculated that the accumulation of C6 aldehydes could be more dependent upon the environment in Muscat Tchervine and Gewürztraminer in comparison with neutral cultivars. As is known, the non-Muscat aromatic varieties contain higher level of terpenes compared with neutral varieties, and this large accumulation of terpenes is considered to be an essential signal with important evolutionary role in pest or disease resistances [24,25,26]. For neutral varieties, the relatively less variation during berry development observed in this study could indicate the indispensable role of hexanal as defense signals in the physiological point of view.

The major C6 alcohols are direct products by alcohol dehydrogenase. These compounds provide green leaf odor for grapes and wines [4,27]. Meanwhile, they are direct precursors for the formation of C6 esters in wines [5]. The ratio of C6 alcohols and aldehydes was used as a marker for grape aromatic maturity according to previous reports [4,28]. In this work, both (*Z*)-3-hexen-1-ol and hexanol were the most abundant among all C6 alcohols especially for 2010 vintage (Table S1). A certain level of (*E*)-2-hexen-1-ol was also detected in various stages and varieties. This showed a little differences from a previous research speculation that little enzymatic activity in transforming (*E*)-2-hexenal to (*E*)-2-hexenol led to rather low level of (*E*)-2-hexen-1-ol in Riesling and Cabernet Sauvignon grapes [7]. The most remarkable difference in the C6 alcohols accumulating pattern among varieties were observed in (*Z*)-3-hexen-1-ol and hexanol. The concentration of (*Z*)-3-hexen-1-ol gradually decreased in the development of Gewürztraminer and Chardonnay, which was in consistent with the pattern of Chardonnay in another study [29] and roughly correspondent with Riesling in a previous report [7]. However, this compound increased with berry development in Syrah and Muscat Tchervine grapes. Hexanol gradually accumulated in the process of berry development of Syrah, Muscat Tchervine and Gewürztraminer grapes, which validated the previously observed steady and significant increasing trend of hexanol of Cabernet Sauvignon and Riesling [4]. The concentration of hexanol in Chardonnay grapes showed a decreasing trend as the berries matured, which was in agreement with a previous work [29]. Besides, the concentration of (*E*)-2-hexen-1-ol of all varieties displayed a constant increasing pattern during berry development.

A total of two C6 esters including hexyl acetate and (*Z*)-3-hexenyl acetate with relatively low levels were detected in this work (Table S1). They were mainly accumulated in early developmental stages of Syrah, Gewürztraminer and Chardonnay grapes, especially for (*Z*)-3-hexenyl acetate. A previous literature suggested that (*Z*)-3-hexenyl acetate could be used in characterizing post-fruit set and pre-veraison stage of wine grapes [4]. Comparatively, the evolution pattern of hexyl acetate was not as stable as (*Z*)-3-hexenyl acetate. This compound gradually increased in Syrah grape in the vintage of 2011, differently from what was observed in 2010.

### 2.2. Varietal Effect on the Concentrations of GLVs in Grape Berries by Multivariate Analysis

**Figure 2:**
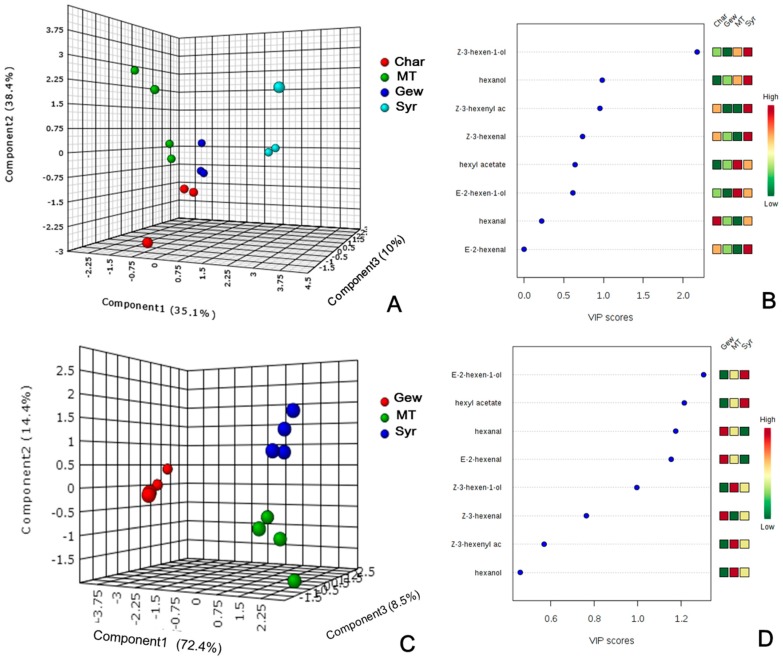
Partial least squares discriminant analysis (PLS-DA) of GLVs from harvested grape berry of different varieties in two vintages. (**A**,**C**) Score plot of 2010 and 2011 respectively; and (**B**,**D**) selected compounds based on Variable Importance in Projection (VIP) scores of 2010 and 2011 respectively.

**Figure 3:**
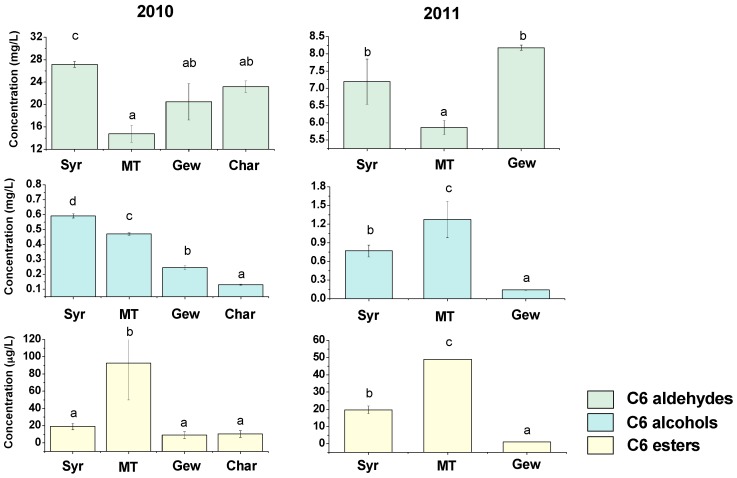
Concentrations of C6 aldehydes, alcohols and esters in four varieties. Different letters among varieties mean significant differences according to Duncan test (*p <* 0.05).

To interpret the variations of GLVs in the four cultivars, partial least squares-discriminate analysis (PLS-DA) was performed by Metabo-Analyst 3.0 [30] on the subset of normalized data of 2010 and 2011 vintage. As displayed in Figure 2A,C, these cultivars were separated from each other, especially Syrah grape. From the Figure 2B, (*Z*)-3-hexen-1-ol, hexanol and (*Z*)-3-hexenyl acetate with a Variable Importance in Projection (VIP) score over 1, contributed to the discrimination of these varieties by first principal component in 2010 vintage. However, (*E*)-2-hexen-1-ol, hexyl acetate, hexanal, (*E*)-2-hexenal and (*Z*)-3-hexen-1-ol were the main C6 compounds discriminating Syrah, Muscat Tchervine and Gewürztraminer grapes in 2011 vintage (Figure 2D).

It can be observed from the bar diagrams (Figure 3) that Syrah berries contained higher concentrations of C6 aldehydes than Muscat Tchervine grapes in both growing seasons. In the 2010 vintage, the concentration of C6 aldehydes of Syrah berries was slightly higher than those of Gewürztraminer and Chardonnay. However, no statistical difference was observed in the 2011 vintage. Regarding C6 alcohols, Muscat Tchervine had relatively higher C6 alcohols than Gewürztraminer in both vintages. Besides, the concentrations of C6 esters in Muscat Tchervine were higher than the other varieties. As we knew, Muscat Tchervine and Gewürztraminer are two aromatic white wine grape cultivars. Terpenes are considered as the most important volatile components determining aroma quality of aromatic varieties. These compounds can provide floral and sweet fruity aroma for the final wine products [14,31]. Until now the reports remain limited regarding the variation and influence of lipid-derived volatiles with green or fruity odor in aromatic grape varieties. Moreover, the effects of lipid-derived volatiles on the grape and wine aroma presentation are also poorly understood even though these C6 volatiles can be detected at a considerable amount in Muscat Tchervine and Gewürztraminer wines [32,33]. The interaction between these compounds contributing to different sensory attributes needs to be investigated. There are several other reports on varietal differences in terms of C6 compounds. It has been demonstrated that Chardonnay skins contained higher amounts of (*E*)-2-hexenal and hexanol than Airén and Macabeo grapes, and Airén grape had the highest level of (*Z*)-3-hexen-1-ol and the lowest level of hexanal and (*E*)-2-hexenal [29]. C6-acetates were considered as the most importantly influenced volatile esters in Shiraz and Cabernet Sauvignon grapes and wines, and a more active LOX pathway in Shiraz grape was suggested then [20]. Thus, based on the results obtained in this work together with previous reports, we concluded that the C6 compounds are also important constituents of varietal typicality.

Table S2 exhibits the *p*-values and F-values obtained by the ANOVA for the three corresponding factors (varieties, maturity and vintage) and their synergy effects. The concentrations of various volatile compounds in grapes from all sampling dates in two vintages were used as variables. The result confirmed that the GLVs concentrations varied significantly with varieties except (*Z*)-3-hexenyl acetate, showing highly variety-dependent. A previous research stated that *(Z)*-3-hexen-1-ol and hexanol were principal components distinguishing different varietal young red wines and their concentrations strongly relied on the variety of grape [19]. Vintage also showed the effect on most of GLVs studied, whereas maturity significantly affected the concentration of C6 aldehydes but not (*Z*)-3-hexen-1-ol and hexyl acetate (Table S2). In addition, both variety and vintage synergistically greatly influenced hexanal, (*E*)-2-hexenal, hexanol, and (*E*)-2-hexen-1-ol, and the interaction of variety and maturity mainly produced effect on C6 alcohols. These results further demonstrated the formers’ findings that both vintage and maturity have noticeable influence on wine grape volatiles [4,28,34,35].

### 2.3. Comparison of Expression of Lipoxygenase-Hydroperoxides lyase (LOX-HPL) Pathway Genes in Different Grape Varieties during Berry Development

**Figure 4:**
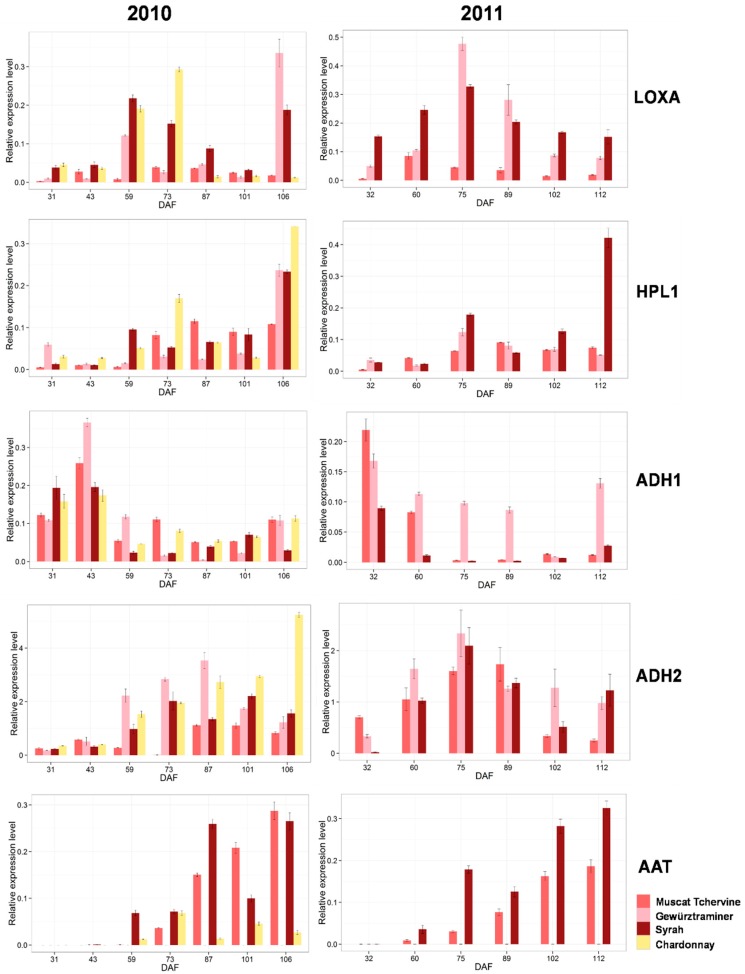
Relative expression level of key genes in LOX-HPL pathway responsible for GLVs synthesis.

C6 volatile compounds are direct products from the LOX-HPL pathway with LOX, HPL, ADH and alcohol acyl transferases (AAT) involved as key enzymes [36]. Figure 4 displays the expression level of major genes involved in GLVs accumulation. Detailed data are displayed in Table S3. Similar expression patterns during grape developing stages among four cultivars with nuances can be observed in these genes (Figure 4). *VvLOXA* and *VvLOXO* are two identified 13-*VvLOXs* in grape berries feasibly responsible for the production of C6 volatiles, and *VvLOXA* was highly expressed in grape skins [8]. The transcript accumulation of *VvLOXA* roughly peaked around veraison and then decreased in Chardonnay and Muscat Tchervine. This result was different from previous observation in which *VvLOXA* expression of Sauvignon Blanc started rising after 50 DAF and remained at a relative higher level after veraison [8]. Despite so, the increasing pattern of *VvLOXA* transcript at pre-veraison was observed in both vintages, suggesting that veraison should be a critical point for the expression of *VvLOXA* in grape berry regardless of varieties. 

HPL1 catalyzes the formation of C6 aldehydes [9]. In this study, *VvHPL1* expressed increasingly during the grape developing process especially in 2010 vintage. The transcript of *VvADH2* was the most abundant among all *VvADHs.* The expression of *VvADH2* presented a gradual increasing trend in developing Chardonnay grapes, which was in accordance with a previous study [37]. However, this gene expression pattern in Chardonnay grapes was totally different from those in other three studied varieties where this gene expression peaked around veraison and decreased in the following developmental stage.

Alcohol acyl transferases (AAT) are able to convert alcohols into volatile esters. In this study a great variance among varieties was observed for *VvAAT* transcript accumulation. The expression abundance of *VvAAT* in Muscat Tchervine and Syrah was the highest, but this gene was expressed at very low level in Gewürztraminer throughout the developing stages. The abundant transcript of *VvAAT* was paralleled with the high concentration of C6 esters in Muscat Tchervine berries, accordingly suggesting that a closely positive correlation existed between *VvAAT* expression and C6 ester accumulation in developing Muscat Tchervine berries.

In this work, the relative expression level of *VvLOXA* in both Gewürztraminer and Syrah of 2010 was at least ten-fold higher than those in Muscat Tchervine and Chardonnay at harvest (Figure 4). Similarly, it expressed at a relative lower level in Muscat Tchervine compared with others in 2011 vintage. The lowest expression level of *VvLOXA* in Muscat Tchervine was in line with its lowest concentration of C6 aldehydes. *VvHPL1* of Chardonnay exhibited the highest expression level among these studied varieties in 2010 vintage, and when compared with Muscat Tchervine, Syrah grapes presented higher *VvHPL1* expression. Similarly, *VvHPL1* of Syrah in 2011 was expressed about eight-fold and five-fold higher than those of Gewürztraminer and Muscat Tchervine, respectively. Considering the fact that Syrah grape contained more abundant C6 aldehydes (Figure 3), we concluded that it was the higher expression level of *VvLOXA* and *VvHPL1* that led to the high accumulation of C6 aldehydes (Figure 4). In addition, the higher expression level of *VvAAT* in Muscat Tchervine was in good agreement with its highest concentration of C6 esters in both vintages. Antalick et al. compared the volatile profiles of Cabernet Sauvignon and Shiraz wines, and suggested that enzymes in LOX pathway may have been more active in Shiraz grapes, thus leading to higher contents of C6 compounds [20]. Our present work supported this suggestion that varietal difference regarding green leaf volatiles should be predominately attributed to different expression level of LOX-HPL pathway genes.

The Pearson’s correlation was calculated with the data of metabolites and relative expression amount of each grape variety from all developing stages. These data were normalized in advance by scale method considering the wide range of the data. As seen in Table 1, the correlation coefficients between the accumulation of GLVs and the expression level of genes from LOX-HPL pathways varied greatly according to varieties. The production of volatiles of Syrah and Gewürztraminer grapes was significantly correlated with the expression of five genes, covering major enzymes in the LOX-HPL pathway; by contrast, in Muscat Tchervine grapes only *VvLOXO*, *VvHPL1* and *VvAAT* transcript abundances highly corresponded to the accumulation of volatiles. The expression of *VvLOXO*, encoding 13-LOX, was negatively correlated with the accumulation of (*E*)-2-hexenal in Chardonnay and Gewürztraminer grape berries, but positively correlated with the production of hexanal in Muscat Tchervine grape. We hypothesized that there possibly existed different regulation mechanisms for *VvLOXO* in different varieties. In Muscat Tchervine grape, a significant correlation was observed between the relative expression of *VvHPL1* and the concentration of (*E*)-2-hexenal. A positive relationship also existed between the expression level of this gene and the concentration of (*E*)-2-hexen-1-ol in Syrah grape. Regarding *ADH2*, it was observed to be positively correlated with (*E*)-2-hexenal production in Chardonnay and Gewürztraminer grape berries. In addition to the result that *AAT* was positively correlated with C6 esters or alcohols in Muscat Tchervine and Syrah, the total concentration of C6 aldehydes was found to be negatively correlated with *AAT* expression in Gewürztraminer, but the correlation was quite opposite in Chardonnay. To sum up, there existed the different correlations between gene expression of LOX-HPL pathway and concentration of C6 compounds among different grape cultivars. The biosynthesis of C6 compounds was complex and differential key genes determining GLVs production were presented in various grape varieties, which needed further research.

## 3. Materials and Methods

### 3.1. Sample Collection

Four *Vitis vinifera* wine grape cultivars: Syrah, Muscat Tchervine, Gewürztraminer and Chardonnay were collected in the same commercial vineyard in Gaotai, Gansu Province in the northwest of China. The vines were cultivated in 2001 and were arranged in north-south oriented rows spaced 2.0 m apart with a distance of about 1.0 m between two plants in each row. The management of the vineyards was in accordance with the local wine grape production technical rules. Grape berries were collected at 18, 32, 44, 60, 74, 88, 102 and 107 DAFs for Syrah and Chardonnay in 2010; 17, 31, 43, 59, 73, 87, 101 and 106 DAFs for Muscat Tchervine and Gewürztraminer in 2010; and 32, 48, 60, 75, 89, 102 and 112 DAFs for Syrah, Muscat Tchervine and Gewürztraminer grapes in 2011 vintage.

Total soluble solids (TSS) were determined with a PAL-79S Digital Hand-held “Pocket” Wine Refractometers (Atago Co., Ltd., Guangzhou, China). pH value was measured by Mettler Toledo FE20 Desktop pH Meter (Mettler Toledo Instruments Co., Ltd., Shanghai, China). The TSS and pH of the grapes are shown in Table 2. Climatic data of the region was obtained from China Meteorological Data Sharing Service System (available at: http://cdc.cma.gov.cn/home.do) and shown in Figure S1.

### 3.2. Extraction and GC-MS Analysis of Volatile Compounds

The extraction and analysis of grape volatile compounds were based on the reported method developed by our research group [21,38,39]. The grape berries with seeds removed were blended with 1 g polyvinylpolypyrrolidone (PVPP) and grounded under continuous liquid nitrogen. After maceration for 4 h at 4 °C, the mixture was centrifuged at 7104× *g* (8000 rpm, 10 cm) at 4 °C for 15 min. Five milliliters of the clear juice with 10 µL internal standard 4-methyl-2-pentanol (4M2P, 1.0018 g/L) and 1 g NaCl added were blended in a 15-mL sample vial tightly capped with a PTFE-silicon septum containing a magnetic stirrer. Afterwards the sample was equilibrated at 40 °C for 30 min while being stirred. Then the pretreated SPME fiber (50/30-μm DVB/Carboxen/PDMS, Supelco, Bellefonte, PA, USA) was inserted into the headspace, extracting for 30 min with samples being continued heating at 40 °C and agitation. The fiber was immediately desorbed for 8 min in the GC injector. The analysis of C6 volatile compounds was performed on an Agilent 6890 GC equipped with Agilent 5975 MS fitted with a 60 m × 0.25 mm HP-INNOWAX capillary column (0.25 μm film thickness) (J & W Scientific, Folsom, CA, USA) as described in previous reports [21]. 

Retention indices of reference standards and mass spectra matching in the standard NIST 08 library were used in the identification of the volatile compounds. A synthetic matrix was prepared in distilled water containing 7 g/L tartaric acid and 200 g/L glucose, pH = 3.3. 4-Methyl-2-pentanol (10 µL) was added before detection as the internal standard. The chemical standards used in this work included hexanal, (*E*)-2-hexenal, hexanol, (*E*)-2-hexen-1-ol, (*Z*)-2-hexen-1-ol, (*E*)-3-hexen-1-ol, (*Z*)-3-hexen-1-ol and hexyl acetate. The standard solution was diluted into eight levels successively and volatile compounds of each level were extracted and analyzed using the same detection method as the grape samples. Standard curves of chemical standards and their lower and upper limit of detection (LOD) were listed in Table S4.

### 3.3. Total RNA Extraction, Purification, cDNA Synthesis and Real-Time qPCR Assay

The RNA extraction, cDNA synthesis and Real-time qPCR methods were followed the previously reported methods [21,40]. Approximately 30 berries were randomly selected and seeds were then removed, and the remaining parts were grounded into powder under continuous liquid nitrogen. Total RNA was isolated from grape berries without seeds using SpectrumTM Plant Total RNA Kit (Sigma-Aldrich, Shanghai, China) according to the protocol. The genome DNA was eliminated by the RNase-free DNase. The quality of the purified RNA was tested by demonstrating the existence of intact ribosomal bands on an agarose gel and A260/A280 by UV spectrophotometer. Then, the corresponding cDNA was synthesized using the qualified RNA as template by Reverse Transcription System Kit (Promega, Madison, WI, USA) according to the instruction.

The relative expression level of genes in LOX-HPL pathway was analyzed by Real-time qPCR using SYBR green method on an ABI 7300 Real-Time System (Applied Biosystems, Foster City, CA, USA) [21]. The difference between the cycle threshold (*C*_t_) of the target gene and the geometric mean of Ct values of reference genes (∆*C*_t_ = *C*_t Target_ − *C*_t RefGene_) was used to obtain the normalized expression of target genes, which corresponds to 2^−∆*C*t^ [41,42]. Ef-α (GenBank accession: EC959059), Actin (GenBank accession: EC969944), and Ubiquitin (GenBank accession: EC929411) were taken as internal controls according previous research [21,40]. Primers for Real-time PCR were designed using PerlPrimer v1.1.19 (available at: http://perlprimer.sourceforge.net/) [21,43]. The dissociation curves of the designed genes were displayed in Figure S2. Two independent extraction procedures were performed for each sample and three technical replications of Real-time qPCR analysis were undertaken.

### 3.4. Statistical Analysis

One-way analysis of variance (ANOVA) and correlation analysis were performed using SPSS 20.0 for windows (SPSS Inc., Chicago, IL, USA). One-way analysis of variance was used to measure differences between means of volatiles concentration employing Duncan’s multiple range tests at a level of *p <* 0.05. Pearson's correlation analysis was performed after data normalization using the “scale” function in the R Base Package. Heat maps of volatile compounds were performed using pheatmap package in R statistical programming language (version 3.1.0) (available at: https://www.r-project.org/) [44]. PLS-DA analysis was performed on MetaboAnalyst 3.0 using “Auto-scaling” in normalization procedure [30]. The concentration of each compound was normalized by dividing the concentration by the maximum value of all samples to enable a comparison of compounds regardless of a wide range of concentrations. A three-way analysis of variance (variety of grape, vintage and sampling dates) was carried out taking the existence of interactions among the factors into account by “Stats” package in R [44].

### 3.5. Reagents and Standards

All the chemical standards used for identification and quantification of GLVs and polyvinylpolypyrrolidone (PVPP) were purchased from Sigma-Aldrich (Shanghai, China). NaCl was purchased from Beijing Chemical Works. SYBR^®^ Premix Ex TaqTM was purchased from TaKaRa Bio (Otsu, Shiga, Japan). The SpectrumTM Plant Total RNA Kit was from Sigma-Aldrich and Reverse Transcription System Kit was purchased from Promega (Madison, WI, USA).

## 4. Conclusions

This study demonstrates the varietal dependence of C6 volatiles, which consequently would have great impact on the sensory quality of wines. C6 profile differences among four distinctive *Vitis vinifera* cultivars had a good association with the expression of LOX-HPL pathway genes, particularly *VvLOXA*, *VvHPL1* and *VvAAT*. High levels of esters corresponded to the high abundance of *VvAAT* in Muscat Tchervine grape. Both high levels of C6 aldehydes and alcohols paralleled with high expression of *VvLOXA* and *VvHPL1* in Syrah grape. The combination of high aldehydes and alcohols with low esters in Gewürztraminer and Chardonnay grapes was consistent with their high transcription levels of *VvLOXA* *or VvHPL1* as well as lower *VvAAT.* The correlation between gene expression and compound accumulation could provide guidance to grape breeders who can link this to parental selection and early generation identification.

## Figures and Tables

**Table 1:** Results of Pearson’s correlation between the concentration of green leaf volatiles (GLVs) and relative expression of lipoxygenase-hydroperoxides lyase (LOX-HPL) genes in four grape varieties.

Genes	Hexanal	(*E*)-2-Hexenal	(*Z*)-3-Hexenal	1-Hexanol	(*Z*)-3-Hexen-1-ol	(*E*)-2-Hexen-1-ol	Hexyl Acetate	(*Z*)-3-Hexenyl Acetate	Sum of C6 Aldehydes	Sum of C6 Alcohols	Sum of C6 Esters
**Chardonnay**											
*LOXO*		**−0.984**	0.811	0.728	**0.962**	−0.757			**−0.865**	0.719	
*ADH1*		−0.746							**−0.868**		
*ADH2*		0.807			−0.761						
*AAT*	0.765								0.741		
**Gewürztraminer**											
*LOXA*						**0.771**					
*LOXO*		−0.550			0.581						
*ADH1*		−0.659			**0.704**						
*ADH2*		**0.856**	−0.653		**−0.667**						
*AAT*		−0.661							−0.639		
**Muscat Tchervine**											
*LOXO*	**0.761**		**0.774**					0.582			0.592
*HPL1*		0.639	−0.653								
*AAT*				0.629			0.568				
**Syrah**											
*LOXA*			−0.574				**−0.662**				
*HPL1*						0.541					
*ADH2*								−0.550			−0.558
*ADH3*			−0.575				−0.638				
*AAT*				0.639	0.654	**0.673**				**0.796**	

Values in bold are significantly correlated at *p* < 0.01. Other values are significantly correlated at *p* < 0.05. Values not significantly correlated at *p* < 0.05 are not shown in this table.

**Table 2:** Total soluble solids (°Brix) and pH of four grape berries (Syrah, Chardonnay, Gewürztraminer and Muscat Tchervine) at all developing stages.

DAF	Muscat Tchervine		Gewürztraminer		Syrah		Chardonnay	
2010	Brix	pH	Brix	pH	Brix	pH	Brix	pH
31	4.4	2.5	3.8	2.6	4.0	2.5	4.0	2.6
43	5.7	2.5	4.8	2.7	4.8	2.5	3.9	2.7
59	8.3	2.6	8.2	2.9	5.1	2.7	10.6	2.8
73	19.5	3.0	15.9	3.0	13.8	2.9	18.5	3.2
87	17.5	2.9	18.8	3.0	16.5	3.2	15.8	3.1
101	20.4	3.0	22.5	3.2	19.3	3.3	20.9	3.4
106	28.1	3.2	27.6	3.3	21.6	3.3	20.1	3.3
2011	Brix	pH	Brix	pH	Brix	pH	-	-
32	5.8	2.5	4.5	2.6	5.2	2.4	-	-
48	6.7	2.5	6.9	2.6	5.6	2.5	-	-
60	11.7	2.6	14.0	2.9	8.3	2.5	-	-
75	18.7	3.0	17.5	3.2	16.9	2.9	-	-
89	19.1	3.1	21.3	3.2	17.5	3.2	-	-
102	24.2	3.2	18.4	3.1	21.4	3.3	-	-
112	28.1	3.2	25.8	3.5	23.6	3.3	-	-

DAF, days after flowering.

## Supplementary Materials

Supplementary materials can be found at www.mdpi.com/1422-0067/17/11/1924/s1.
