# Supplementary material for: Varietal Dependence of GLVs Accumulation and LOX-HPL Pathway Gene Expression in Four Vitis vinifera Wine Grapes
Source: Int J Mol Sci. 2016 Nov 23;17(11):1924. doi: 10.3390/ijms17111924 (PMC5133920; doi:10.3390/ijms17111924)
Supplement: Supplementary file 1 [file ijms-17-01924-s001.zip › ijms-148316(suppl.)Figures.pdf]

Supplementary Materials: Varietal Dependence of GLVs Accumulation and LOX-HPL Pathway Gene Expression in Four *Vitis vinifera* Wine Grapes

Xu Qian, Xiao-Qing Xu, Ke-Ji Yu, Bao-Qing Zhu, Yi-Bin Lan, Chang-Qing Duan and Qiu-Hong Pan

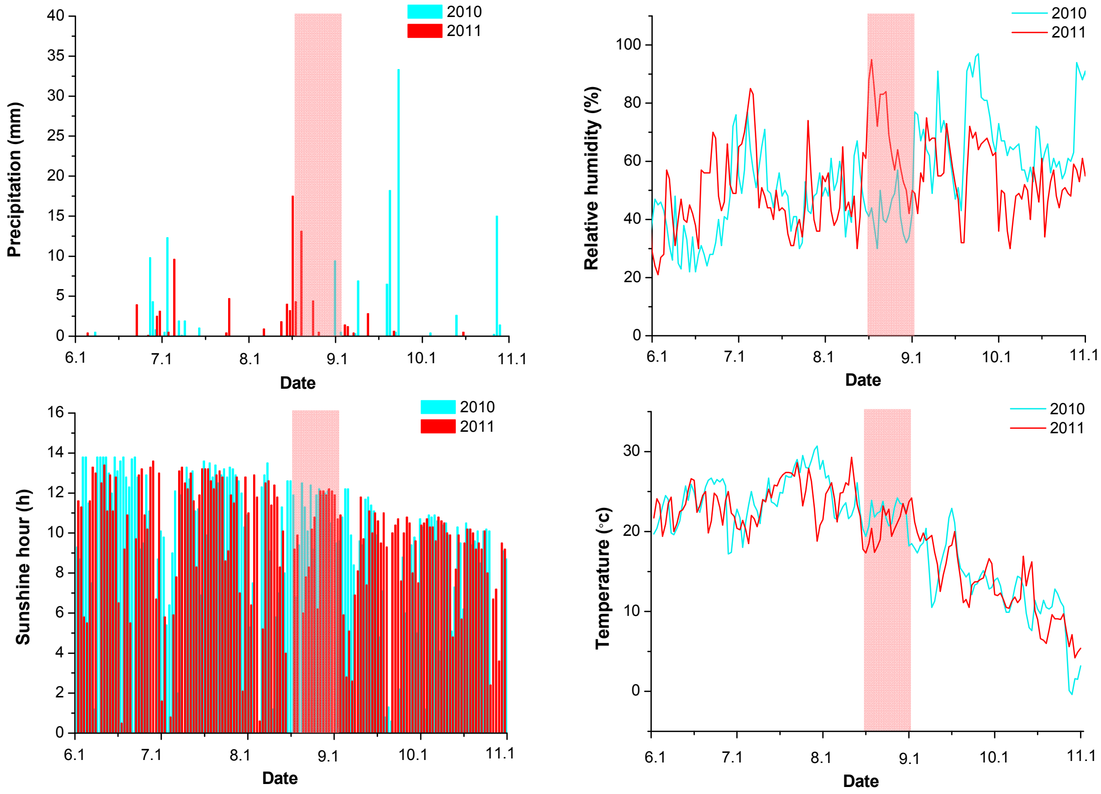

Figure S1. Meteorological data (precipitation, relative humidity, sunshine hour and temperature) during grape berry development of 2010 and 2011 in Gaotai, Gansu Province, China. Pink bars represent veraison stage of grapes.

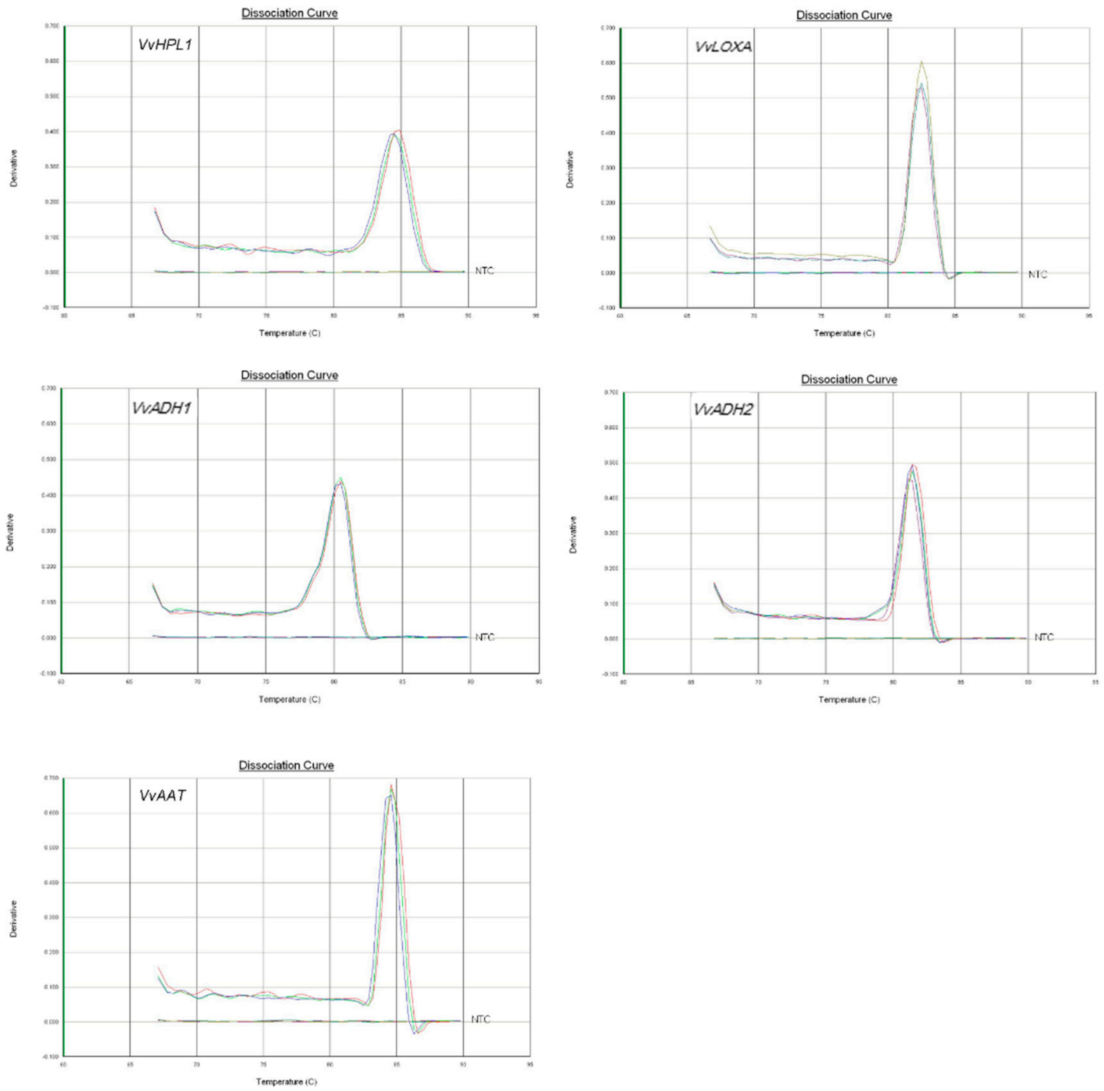

Figure S2. Dissociation curves of designed primers for *VvLOXA*, *VvHPL1*, *VvAAT*, *VvADH1* and *VvADH2*. NTC: non tempelate control.
